# Age-Related Changes in the Composition of Gut *Bifidobacterium* Species

**DOI:** 10.1007/s00284-017-1272-4

**Published:** 2017-06-08

**Authors:** Kumiko Kato, Toshitaka Odamaki, Eri Mitsuyama, Hirosuke Sugahara, Jin-zhong Xiao, Ro Osawa

**Affiliations:** 1 Next Generation Science Institute R&D Division, Morinaga Milk Industry Co., Ltd., 5-1-83, Higashihara, Zama-city, Kanagawa 252-8583 Japan; 2 Department of Bioresource Science, Graduate School of Agricultural Science, Kobe University, Kobe, Hyogo Japan

## Abstract

Bifidobacteria are one of the major components in human microbiota that are suggested to function in maintaining human health. The colonization and cell number of *Bifidobacterium* species in human intestine vary with ageing. However, sequential changes of *Bifidobacterium* species ranging from newborns to centenarians remain unresolved. Here, we investigated the gut compositional changes of *Bifidobacterium* species over a wide range of ages. Faecal samples of 441 healthy Japanese subjects between the ages of 0 and 104 years were analysed using real-time PCR with species-specific primers. *B. longum* group was widely detected from newborns to centenarians, with the highest detection rate. *B. breve* was detected in approximately 70% of children under 3 years old. *B. adolescentis* and *B. catenulatum* groups were predominant after weaning. *B. bifidum* was detected at almost all ages. The detection rate of *B. dentium* was higher in the elderly than in other ages. *B. animalis* ssp. *lactis* was detected in 11.4% of the subjects and their ages were restricted. *B. gallinarum* goup was detected in only nine subjects, while *B. minimum* and *B. mongoliense* were undetected at any age. The presence of certain *Bifidobacterium* groups was associated with significantly higher numbers of other *Bifidobacterium* species/subspecies. Inter-species correlations were found among each species, exception for *B. animalis* ssp. *lactis*. These results revealed the patterns and transition points with respect to compositional changes of *Bifidobacterium* species that occur with ageing, and the findings indicate that there may be symbiotic associations between some of these species in the gut microbiota.

**Electronic supplementary material:**

The online version of this article (doi:10.1007/s00284-017-1272-4) contains supplementary material, which is available to authorized users.

## Introduction

Members of the genus *Bifidobacterium* naturally inhabit human gastrointestinal tract (GIT), and are thought to play pivotal roles in maintaining human health [4, 12]. *Bifidobacterium* is the most predominant genus of the breast-fed infant gut microbiota. However, the numbers of this genus substantially decrease after weaning and continue to decrease with age [33, 40, 44]. In addition, bifidobacterial composition at species level was reported to vary between life stages. To date, approximately ten species/subspecies *Bifidobacterium* have been found in human intestine. Previous reports have shown that *Bifidobacterium breve* and *B. longum* are the predominant species of the infant intestinal microbiota [2, 16]. Avershina et al. [1] evidently demonstrated that a switch occurs from a childhood- to an adult-associated microbial profile between one and two years of age. They further suggested that this change was driven by the keystone of *B. breve* strains. *Bifidobacterium breve* was highly prevalent in infant gut microbiota population during the first year of life, and showed negative correlation with *B. longum* in the adult population.

In contrast to the infant gut, the adult gut microbiota is predominated by *B. adolescentis* and *B. catenulatum* groups, in addition to the infant-associated *B. longum* ssp. *longum* [1, 8, 15]. These adult-type predominant *Bifidobacterium* species persisted in elderly, with an increase in the prevalence of *B. dentium* species [8, 21, 28]. Recently, Wang et al. [39] revealed that the faecal *Bifidobacterium* species in centenarians of Chinese descent were different from those of young elderlies. Despite the common faecal *Bifidobacterium* species, including *B. dentium*, *B. longum* ssp. *longum*, *B. thermophilum*, members of the *B. catenulatum* group, and *B. adolescentis* in younger elderlies and centenarians, centenarians were reported to possess certain unique species, such as *B. minimum*, *B. gallinarum/B. pullorum*/*B. saecularmay* and *B. mongoliense*, which were absent in the younger elderlies [39].

Although some studies have compared the *Bifidobacterium* species present in healthy adults to those in infants and/or elderly individuals [8, 10, 41], no reports have examined the sequential changes that occur in *Bifidobacterium* species during life spans ranging from newborns to centenarians.

The aim of this study was to improve the current understanding of the compositional changes of *Bifidobacterium* species along with ageing. Here, we investigated the sequential changes that occur in the composition of *Bifidobacterium* species in 441 healthy Japanese subjects over a wide age range, including individuals from 0 to 104 years old.

## Materials and Methods

### Subject

Four hundred forty-six faecal samples were collected from 441 community-dwelling Japanese volunteers (essentially one sample per time point per subject, except for two samples that were collected from two boys and one from one girl at only pre-weaning and weaning and three samples from one girl at pre-weaning, weaning and 5 years old) between 0 and 104 years of age (180 men, 261 women). Subjects aged over 80 years were recruited and confirmed to be community dwellers. Faecal samples were collected from subjects who participated in three different studies. Two study protocols [25, 26] included the collection of faeces from subjects aged 21 to 65 years or from community-dwelling elderly individuals, were approved by the Local Ethics Committee of the non-profit organization Japan Health Promotion Supporting Network (Wakayama, Japan). The third study protocol [25] including the collection of faeces from subjects aged 0 to 100 years old was approved by the ethical committee of the Kensyou-kai Incorporated Medical Institution (Osaka, Japan). Written and informed consent was obtained from all subjects or their legal guardians or relatives. The subjects were divided into 10-year age groups according to their age, except for subjects who were younger than 10 years old. Infants and children were divided into four groups: pre-weaning, weaning, weaned to 3 years old and 4–9 years old. The distribution of subjects according to age is shown in Table 1.

**Table 1:** Sample distribution

Group	Age		Number of samples	(Male/female)
	Segmentation	(Mean ± SD)		
1	Pre-weaning	(0.3 ± 0.1)	16	(9/7)
2	Weaning	(0.8 ± 0.4)	12	(7/5)
3	Weaned-3 years old	(2.4 ± 0.6)	21	(10/11)
4	4–9 years old	(5.9 ± 1.8)	17	(7/10)
10	10–19 years old	(14.1 ± 3.6)	10	(7/3)
20	20–29 years old	(25.8 ± 2.7)	42	(16/26)
30	30–39 years old	(34.3 ± 2.5)	114	(54/60)
40	40–49 years old	(43.7 ± 3.1)	37	(13/24)
50	50–59 years old	(53.5 ± 2.7)	29	(13/16)
60	60-69 years old	(64.2 ± 2.9)	42	(14/28)
70	70–79 years old	(75.5 ± 2.9)	31	(12/19)
80	80–89 years old	(83.2 ± 2.4)	51	(17/34)
90	90–99 years old	(94.2 ± 2.7)	19	(4/15)
100	Over 100 years old	(101.6 ± 1.8)	5	(0/5)
	Total	(44.9 ± 27.8)	446	(183/263)

Gut microbiota was analysed in one sample per subject, except for two samples that were obtained from one boy and one girl at pre-weaning and weaning and three samples that were obtained from one girl at pre-weaning, weaning and 5 years of age

### DNA Extraction from Faecal Samples

The collection and storage of faecal samples and DNA extraction were performed using previously described methods [25].

### Real-Time PCR

Real-time PCR was performed using an ABI PRISM 7500 Fast Real-Time PCR system (Thermo Fisher Scientific K.K., Uppsala, Sweden) with SYBR Premix Ex Taq (TaKaRa Bio, Shiga, Japan). Table 2 shows the primer sets that were used in this study. Since *B. longum* ssp. *infantis*, *B. longum* ssp. *longum* and *B. longum* ssp. *suis* are closely related in their 16S rDNA similarity [16, 18, 29], these three species are treated as the members of the *B. longum* group. Similarly, *B. catenulatum* and *B. pseudocatenulatum* are detected as the members of *B. catenulatum* group. *B. adolescentis* consisting genotypes A and B are detected with the primer sets for *B. adolescentis* group. Primer sets for *B. gallinarum group, B. mongoliense* and *B. minimum* were designed based on the sequences of a house keeping gene, *clpC,* with the sequences of related species/subspecies acquired from GenBank (http://www.ncbi.nlm.nih.gov/). After multiple alignments by MEGA 6 [31], unique regions were selected as a target for the specific primers. Since the genomic information of *B. gallinarum*, *B. pullorum* and *B. saeculare* is closely related [11], these three species are treated as *B. gallinarum* group and the primer set for *B. gallinarum* is designed to detect these three species.

**Table 2:** The PCR primers used to detect each species

Target species	Primer	Sequence (5′ to 3′)	Reference
*Bifidobacterium adolescentis* group	BiADOg-1a	CTCCAGTTGGATGCATGTC	[16]
	BiADOg-1b	TCCAGTTGACCGCATGGT	
	BiADOg-2	CGAAGGCTTGCTCCCAGT	
*Bifidobacterium animalis* ssp. *lactis*	Bflact2	GTGGAGACACGGTTTCCC	[37]
	Bflact5	CACACCACACAATCCAATAC	
*Bifidobacterium bifidum*	BiBIF-1	CCACATGATCGCATGTGATTG	[17]
	BiBIF-2	CCGAAGGCTTGCTCCCAAA	
*Bifidobacterium breve*	BiBRE-1	CCGGATGCTCCATCACAC	[17]
	BiBRE-2	ACAAAGTGCCTTGCTCCCT	
*Bifidobacterium catenulatum* group	BiCATg-1	CGGATGCTCCGACTCCT	[17]
	BiCATg-2	CGAAGGCTTGCTCCCGAT	
*Bifidobacterium dentium*	BiDEN-1	ATCCCGGGGGTTCGCCT	[16]
	BiDEN-2	GAAGGGCTTGCTCCCGA	
*Bifidobacterium gallinarum* group	BiGall_clpC_F2	TGTGACGATCACCGATGC	This study
	BiGall_clpC_R2	GCTTGTGCAGCTCGCTCT	
*Bifidobacterium longum* group	BiLONg-1	TTCCAGTTGATCGCATGGTC	[17]
	BiLONg-2	TCSCGCTTGCTCCCCGAT	
*Bifidobacterium longum* ssp. *longum*	BiLON-1	TTCCAGTTGATCGCATGGTC	[16]
	BiLON-2	GGGAAGCCGTATCTCTACGA	
*Bifidobacterium minimum*	BiMini_clpC_F	GGTCTTCGCAGCCGGTAT	This study
	BiMini_clpC_F	CGACAACCATGCTGACGTTC	
*Bifidobacterium mongoliense*	BiMong_clpC_F	ACGTGACCATCACCGACAAG	This study
	BiMong_clpC_R	CATCTTCACATCGGAACCAC	

The amplification programme consisted of one cycle of 95 °C for 20 s, then 40 cycles of 95 °C for 20 s, 55 °C for 20 s and 72 °C for 50 s, and one final cycle of 95 °C for 15 s. The fluorescent product was detected in the last step of each cycle. Following amplification, a melting temperature analysis of the PCR products was performed to determine the specificity of the PCR. Melting curves were obtained by slow heating at 0.2 °C/s increments from 60 to 95 °C, with continuous collection of fluorescence data. To determine the number of *Bifidobacterium* species that were present in each sample, we compared the results to a standard curve that was generated using standard DNA in the same experiment. The following *Bifidobacterium* species were used as the standard strains for species-specific quantification: *B. adolescentis* JCM1275^T^, *B. animalis* ssp. *lactis* JCM1190^T^, *B. bifidum* JCM1255^T^, *B. breve* JCM1192^T^, *B. dentium* JCM1195^T^, *B. gallinarum* JCM6291^T^, *B. longum* ssp. *longum* JCM1217^T^, *B. minimum* JCM5821^T^, *B. mongoliense* JCM 15461^T^ and *B. pseudocatenulatum* JCM1200^T^.

### Statistical Analyses

All analyses were performed using the IBM SPSS Statistics, version 22.0, statistical software package (IBM Corp., Armonk, NY, USA). Intergroup differences in the number of species were analysed using unpaired t-tests. Spearman’s correlation coefficient was used to determine the relationships among *Bifidobacterium* species by substituting the data with 6 log_10_ for individuals under the detection limits. For all statements, *P* values < 0.05 were considered statistically significant.

## Results

### Quantitative PCR Detection of *Bifidobacterium* Species

**Fig. 1:**
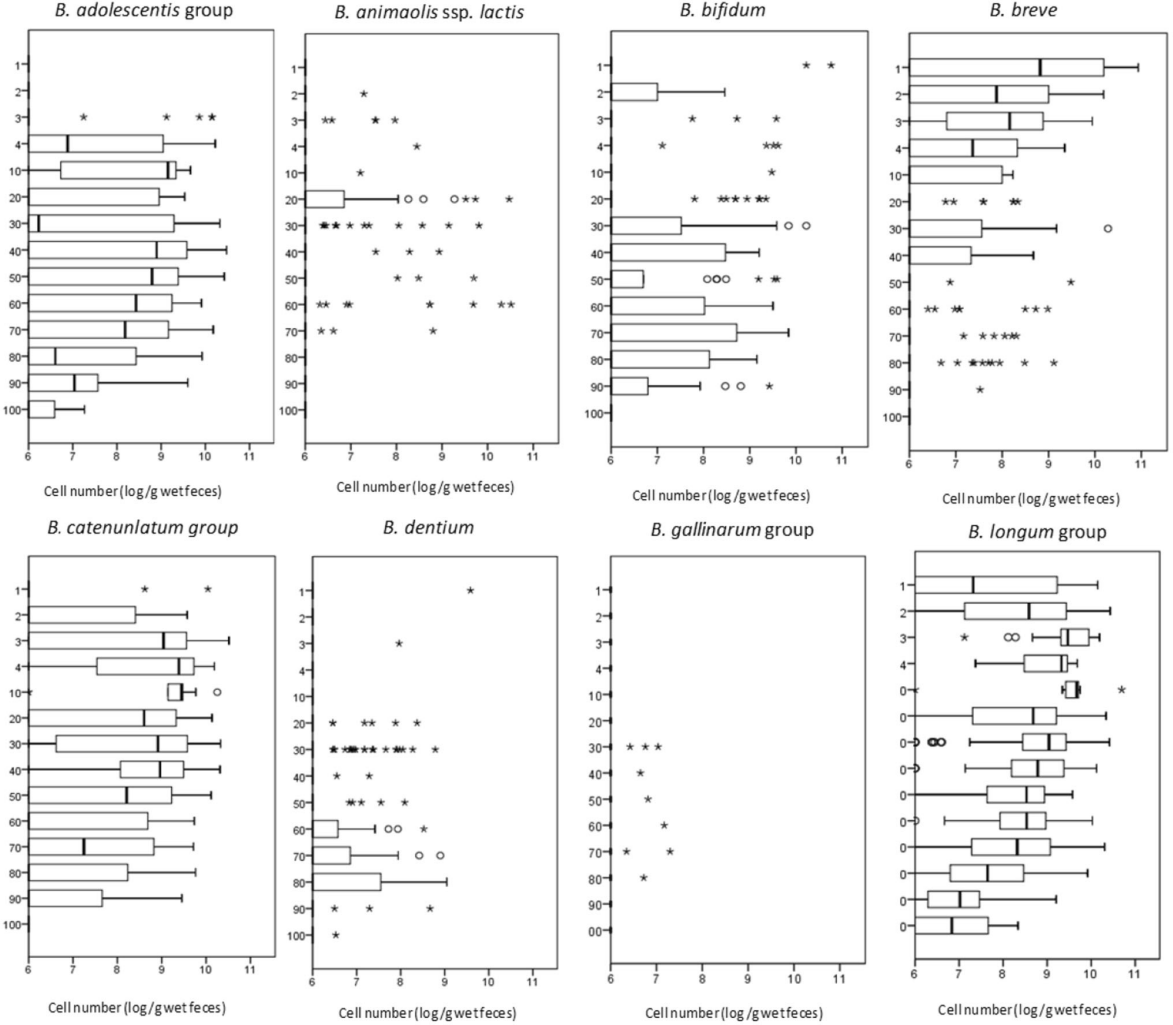
Distribution of seven *Bifidobacterium* species in the faeces of healthy Japanese subjects aged 0–104 years. Cell numbers were determined as the log10 of cells per gram wet weight of faecal samples. The box plots show the interquartile range (IQR) of the cell numbers shown in each section. *Open circles* and *asterisks* indicate outliers between 1.5- and 3.0-fold IQR and over 3.0-fold IQR, respectively. The age of each group is as shown in Table 1. The detection limits for each bacterial species were determined using real-time PCR to be 10^6^/g wet weight of faeces

**Fig. 2:**
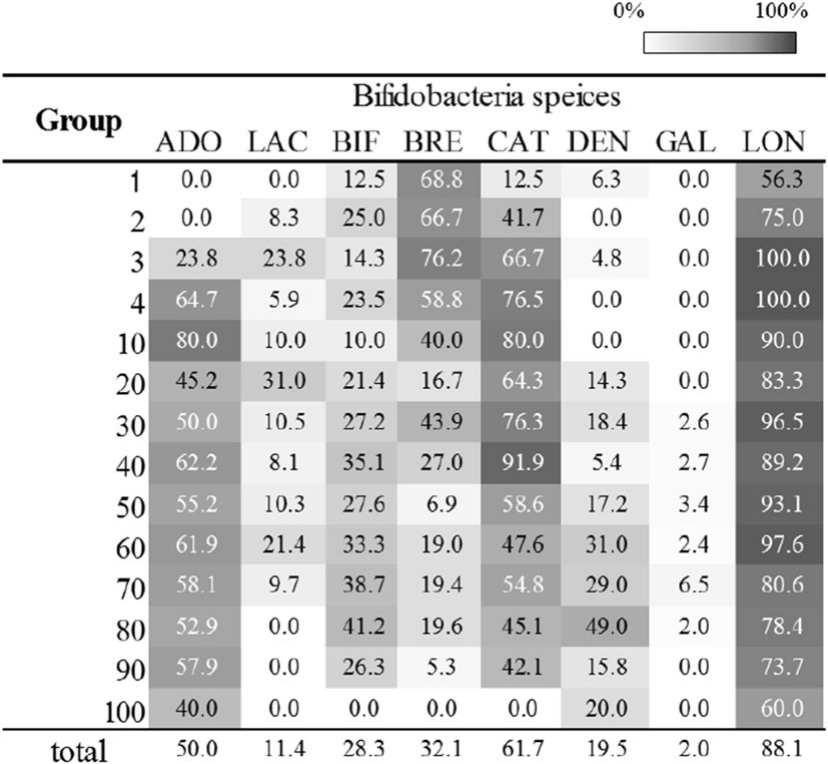
Detection rate of each bifidobacteria species in each segmented age group. ADO, *B. adolescentis* group; LAC, *B. animalis* spp. *lactis*; BIF, *B. bifidum*; BRE, *B. breve*; CAT, *B. catenulatum* group; DEN, *B. dentium*; GAL, *B. gallinarum* group;LON, *B. longum* group. The age of each group is as shown in Table 1

Figures 1 and 2 show the distributions and detection rates, respectively, of the eight *Bifidobacterium* species that were found in the faeces of 441 subjects. *B. longum* group was widely detected (8.7 ± 0.9 log_10_ cells per gram of wet faeces) in the majority of individuals, from infants before weaning to centenarians, and its detection rate was the highest of the investigated species (88.1%). The distribution of *B. longum* ssp. *longum* was nearly equal to those of *B. longum* group (Online Resource 1). *B. catenulatum* group species and *B. bifidum* were also commonly detected at all ages except for the centenarians, but their detection rates were lower (61.7 and 28.3%, respectively) than the rate for *B. longum* group. The *B. catenulatum* group and the *B. adolescentis* group were predominant after weaning. Conversely, *B. breve* was detected in 71.4% of children under 3 years old. The number of *B. breve* cells decreased with age, although its detection rate was consistently high in individuals younger than 10 years old. *B. dentium* was detected in some of the subjects who were over 20 years old, and its detection rate increased until the subjects reached 90 years old. *B. animalis* ssp. *lactis*, which is not considered a species of the human gut microbiota, was also detected in 11.4% of the subjects, but it was restricted to individuals between weaning and less than 80 years old. Both *B. dentium* and *B. animalis* ssp. *lactis* were present as relatively small populations of 7.4 ± 0.7 and 7.9 ± 1.2 log_10_ cells per gram of wet faeces, respectively. *B. gallinarum* goup was detected in only nine subjects aged 31–84 years, while *B. minimum* and *B. mongoliense* were not detected at any age.

### Differences in *Bifidobacterium* Species Across Individuals

**Fig. 3:**
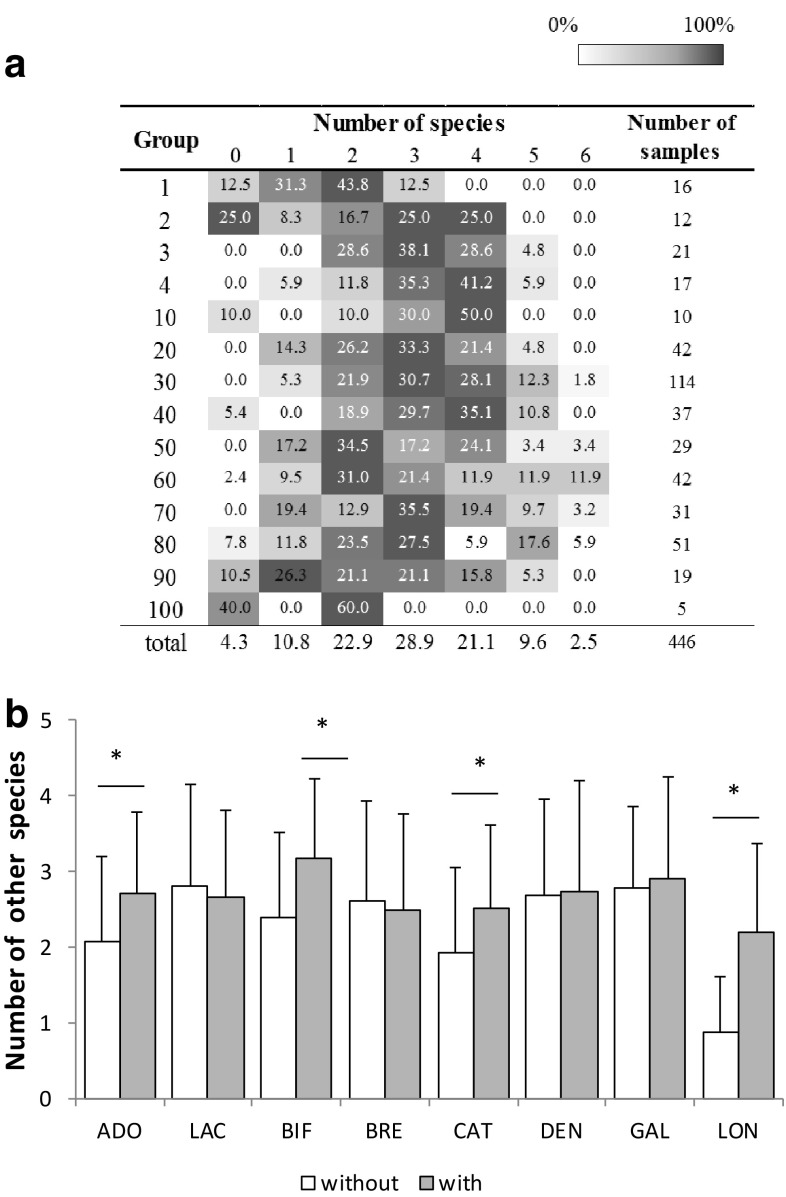
Number of *Bifidobacterium* species. **a** Distribution of the numbers of *Bifidobacterium* species that were found in individuals in each age group. The age of each group is as shown in Table 1. **b** Differences in species numbers between subjects with or without the presence of identical species (i.e. the number of species excluding the identical species). ADO, *B. adolescentis* group; LAC, *B. animalis* spp. *lactis*; BIF, *B. bifidum*; BRE, *B. breve*; CAT, *B. catenulatum* group; DEN, *B. dentium*; GAL, *B. gallinarum* group; LON, *B. longum* group

**Fig. 4:**
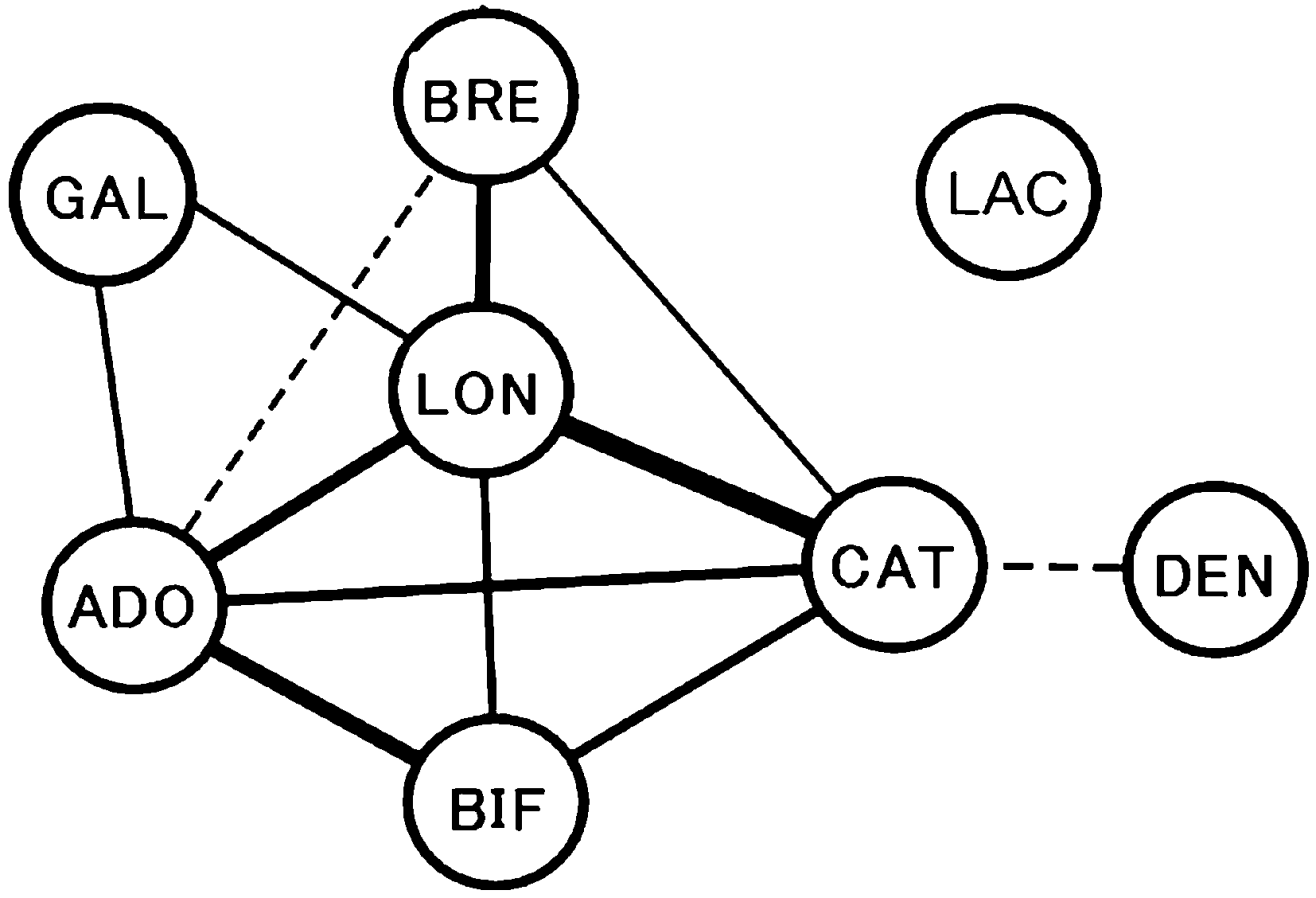
Possible ecological compatibility among *Bifidobacterium* species. The *solid* and *dashed lines* indicate positive and negative correlations, respectively. The widths of the connected lines indicate the abundance of the coefficient value. ADO, *B. adolescentis* group; LAC, *B. animalis* spp. *lactis*; BIF, *B. bifidum*; BRE, *B. breve*; CAT, *B. catenulatum* group; DEN, *B. dentium*; GAL, *B. gallinarum* group; LON, *B. longum* group. Online Resource 1. Distribution of *B. longum* ssp. *longum* in the faeces of healthy Japanese subjects aged 0–104 years. The age of each group is as shown in Table 1

We then investigated the number of species that were detected in each individual. The mode for the species number was 2 to 4 in almost all segmented age groups (Fig. 3a). We found that the presence of *B. longum* group, *B. adolescentis* group, *B. catenulatum* group or *B. bifidum* led to the detection of significantly higher numbers of other *Bifidobacterium* species (Fig. 3b). The difference was the largest between subjects with (2.18 ± 1.17) or without (0.92 ± 0.75) *B. longum* group. A correlation analysis revealed that the presence of *B. longum* group was significantly associated with the presence of *B. catenulatum* group (*r* = 0.414, *P* < 0.001), *B. breve* (*r* = 0.320, *P* < 0.001), *B. adolescentis* group (*r* = 0.239, *P* < 0.001), *B. bifidum* (*r* = 0.139, *P* = 0.003) and *B. gallinarum* group (*r* = 0.123, *P* = 0.009, Fig. 4). *B. breve*, which was defined as an infant-type species, showed the significant negative correlation with *B. adolescentis* group species (*r* = −0.124, *P* = 0.009), which were defined as adult-type species. *B. adolescentis* group was significantly associated with *B. gallinarum* group (*r* = 0.145, *P* = 0.002) and *B. catenulatum* group (*r* = 0.119, *P* < 0.001). Another significant negative correlation was observed in *B. dentium*, which was defined as an elderly type species, and *B. catenulatum* group species (*r* = −0.142, *P* < 0.001). *B. animalis* ssp. *lactis* was not significantly correlated with other *Bifidobacterium* species.

## Discussion

The composition of the gut microbiota changes with age [22]. Recent studies using molecular methods have also indicated that there are clear differences in the composition of the gut microbiota between infants, toddlers, adults and the elderly [3]. Some reports have shown that there are intermittent differences between age groups [3, 8, 27, 41]. However, few reports have investigated the long-term, sequential changes in the composition of gut microbiota. Recently, we showed that there are sequential changes in the composition of the gut microbiota at the genus level in Japanese subjects over a wide range of ages, from 0 to 104 years old [25]. In this study, we focused on the genus *Bifidobacterium* and investigated the details of the changes that occurred in these species at the species level over the study age range. Our results are in agreement with those of previous studies indicating that there are differences in the proportions and types of *Bifidobacterium* species in the gut microbiota between infants, adults and the elderly [1, 8, 41].

Our results show the patterns and transition points that occur with ageing in the prevalence of each *Bifidobacterium* species. There is a decrease in the cell numbers of *B. breve* in subjects over 50 years old. Conversely, there is an increase in the cell numbers of *B. dentium*, a bacteria species whose genomic sequence has been reported to reflect its adaptation to human oral cavities [38], in subjects over 60 years old. We have found that certain oral bacteria that have been suggested to have difficulty in reaching the intestinal tract due to the GIT barriers, such as gastric juices and bile acid, were enriched in the elderly gut microbiota [25]. Our data imply that the decline in GIT functionality in the elderly might lead to significant changes in the presence of *B. dentium* and other oral bacteria in the gut.

*Bifidobacterium longum* group was the major species that were detected in all segmented age groups. Among *B. longum* group, *B. longum* ssp. *longum* and *B. longum* ssp. *infantis* have been reported to be detected in human gut microbiota [14, 16]. The distribution of *B. longum* ssp. *longum* was nearly equal to those of *B. longum* group, suggesting that the majority of *B. longum* group in human gut microbiota was *B. longum* ssp. *longum* at least in this study. Some strains of *B. longum* ssp. *longum* were shown to be genetically equipped to utilize both plant-derived and human milk oligosaccharide (HMO)-derived sugars [24, 42]. This characteristic might explain why *B. longum* ssp. *longum* is a widespread species at all ages. We also tried to evaluate the distribution of *B. longum* ssp. *infantis*, which has been reported to be a champion colonizer from the perspective of genomic information [36] and was frequently found in infants [16, 21]. However, we failed to enumerate them precisely due to a cross-reactivity of the reported specific primers, which were designed based on the sequence of type strain *B. longum* ssp*. infantis* [16, 30], against some strains of *B. breve* (data not shown). In addition, recent studies based on genomic information have indicated some mismatches between phenotype and genotype property, e.g. *B. longum* ssp. *infantis* 157 F and CCUG 52,486, which are assigned to *infantis* subspecies based on the phenotypes but are genetically *longum* subspecies [23]. Future studies such as taxonomical investigation as well as development of primers with high specificity are necessary to accurately estimate the cell numbers of *B. longum* ssp. *infantis* in the human gut microbiota.

*B. animalis* ssp. *lactis*, which is not considered a species of the human gut microbiota [35], was also detected in 11.4% of the subjects. Because this taxon is predicted to have a limited number of hypothetical glycosyl hydrolases/carbohydrate pathways [19, 20, 24], it seems to be unsuitable for using the carbon resources in the human gut environment. However, because *B. animalis* ssp. *lactis* is commonly applied as a probiotic in dairy products, its detection might be a reflection of food intake such as yogurt. Further identification of the *B. animalis* ssp. *lactis* strain found in the subjects and/or information regarding the dietary habits of the subjects would clarify the reason of this finding. The correlation analysis indicated that all of the species investigated in this study, with the exception of *B. animalis* ssp. *lactis*, were significantly correlated with other species. These results also suggest that *B. animalis* ssp. *lactis* is not a commensal component in the human gut. Previous study reported the detection of *B. gallinarum/B. pullorum*/*B. saecularmay, B. minimum* and *B. mongoliense* in Chinese centenarian [39]. However, these species were not detected in the five Japanese centenarians in the present study.

We found that subjects in whom *B. longum* group, *B. bifidum*, *B. adolescentis* group or *B. catenulatum* group species were detected to possess a significantly higher number of other *Bifidobacterium* species, suggesting that there may be symbiotic associations between *Bifidobacterium* species. *B. breve* was reportedly to be capable of cross-feeding on sugars released during the mucin-degrading activity of *B. bifidum*, which can utilize host-derived glycans, including those present in mucin [34] or HMOs [5], via two secreted N-acetyl-β-glucosaminidases and two secreted sialidases, respectively [20]. In a clinical study, Tannock et al. [32] also reported that breast milk-fed infants who harboured *B. bifidum* comprising greater than 10% of the total microbiota, had the highest abundances of total bifidobacteria. Interestingly, Ferrario et al. [7] found that co-cultivating *Bifidobacterium* species in media that simulated the adult and infant human gut environments resulted in an increase in the transcription of key genes required for biosynthesis of exopolysaccharides, which have been reported to have some important roles for modulating various aspects of bifidobacterial–host interaction, including the ability of commensal bacteria to remain immunologically silent and in turn provide pathogen protection [6]. However, it is still unclear whether these positive relationships are consistent among the *Bifidobacterium* species studied in this study as it is possible that these species which possess a positive correlation might just grow well under the same environmental conditions without sharing a mutualistic relationship. The results of our statistical analysis must therefore be interpreted cautiously because they are based on a limited dataset. An advanced culture method is necessary to clarify the relationships between each set of *Bifidobacterium* species.

Similar to what was found in a previous report [16], we found that the average number of species detected per individual was two and three in infants and adults, respectively. The average number of species was not different between adults and the elderly, until they reached 81–90 years old, at which point the number was slightly decreased with age in individuals over 91 years old. The average number of species and the cell numbers of each species in centenarians were relatively lower, but *B. longum* group, *B adolescentis* group and *B. dentium* were detected in centenarians. In previous reports, *B. longum* ssp. *longum* and *B. adolescentis* group were isolated from Chinese centenarians [9, 13], and these species have been suggested to improve intestinal digestion and the ability to enhance immune-barrier functions in mouse models [43]. These species may have important functions that contribute to the maintenance of a healthy condition in centenarians.

In conclusion, we provide a picture of the changes of *Bifidobacterium* species that occur with ageing in the gut microbiota. Our results indicate that there are patterns and transition points with respect to age-related changes in the composition of *Bifidobacterium* species. The positive correlations of our findings highlighted that there may be symbiotic associations between human residential species.

Our findings contribute to clarifying what is known about the composition of bifidobacteria in the gut of healthy populations at each age period. Further analyses that investigate genomic information and in vitro co-cultivation experiments would be valuable for evaluating the relationships between *Bifidobacterium* species.

## Electronic Supplementary Material

Below is the link to the electronic supplementary material.

Supplementary material 1 (PPTX 110 kb)

Supplementary material 2 (DOC 31 kb)
