# Supplementary material for: Age-Related Changes in the Composition of Gut Bifidobacterium Species
Source: Curr Microbiol. 2017 Jun 8;74(8):987–95. doi: 10.1007/s00284-017-1272-4 (PMC5486783; doi:10.1007/s00284-017-1272-4)
Supplement: Supplementary file 1 — Supplementary material 1 (PPTX 110 kb) [file 284_2017_1272_MOESM1_ESM.pptx]

## Slide 1
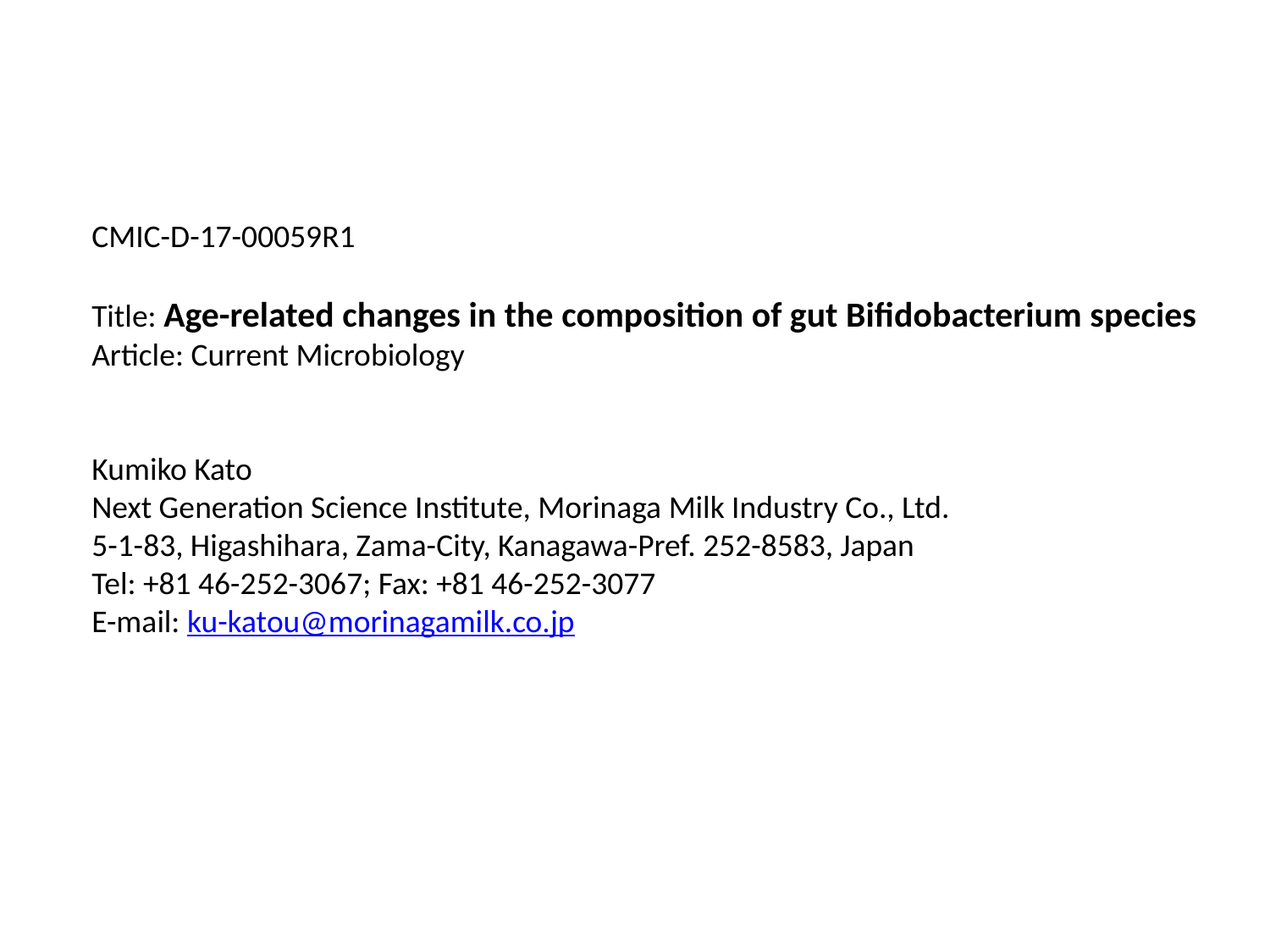

CMIC-D-17-00059R1
Title: Age-related changes in the composition of gut Bifidobacterium species
Article: Current Microbiology
Kumiko Kato
Next Generation Science Institute, Morinaga Milk Industry Co., Ltd.
5-1-83, Higashihara, Zama-City, Kanagawa-Pref. 252-8583, Japan
Tel: +81 46-252-3067; Fax: +81 46-252-3077
E-mail: ku-katou@morinagamilk.co.jp

## Slide 2
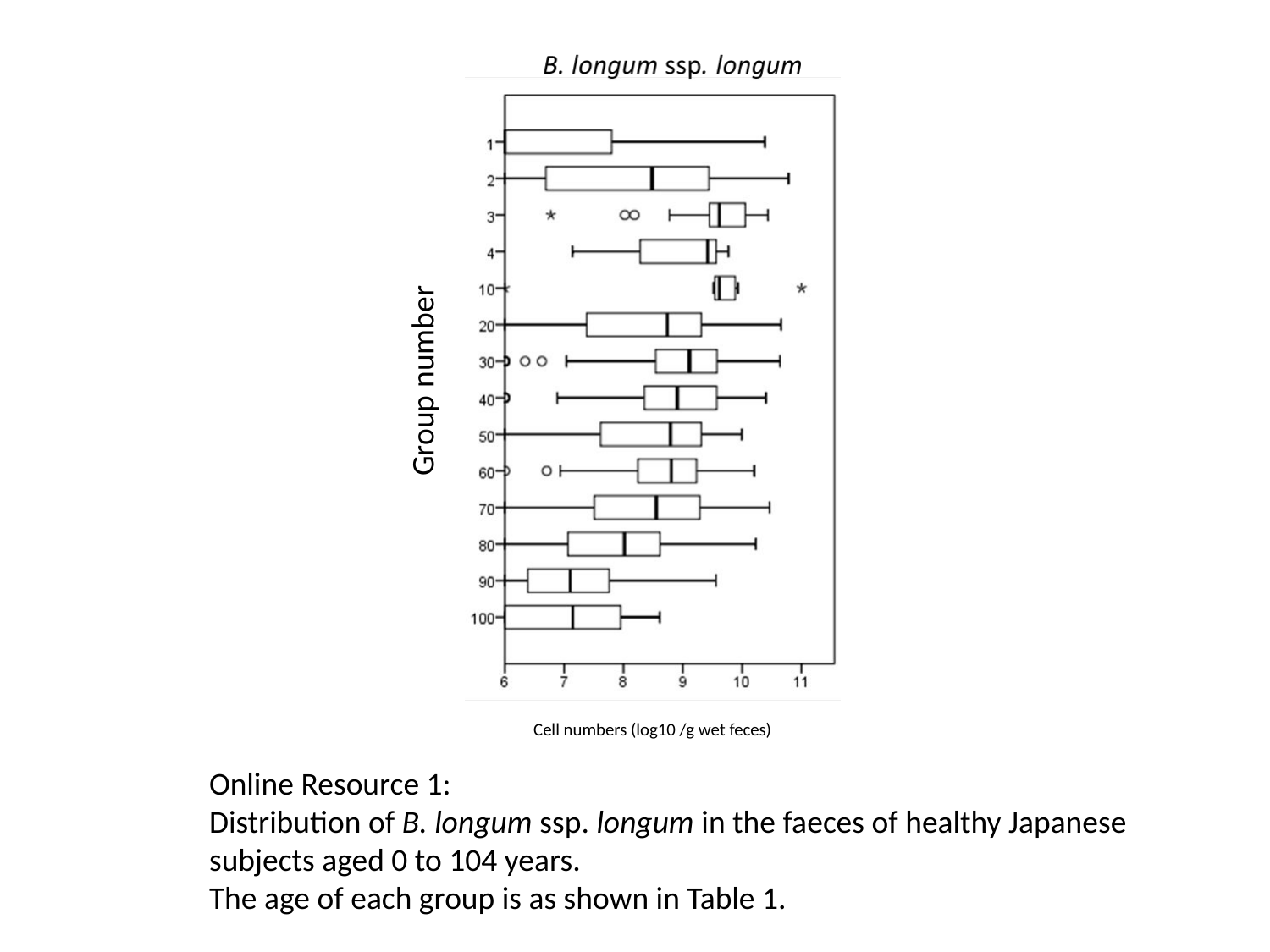

Group number
Cell numbers (log10 /g wet feces)
Online Resource 1:
Distribution of B. longum ssp. longum in the faeces of healthy Japanese subjects aged 0 to 104 years.
The age of each group is as shown in Table 1.
