# Supplementary material for: Age-Related Changes in the Composition of Gut Bifidobacterium Species
Source: Curr Microbiol. 2017 Jun 8;74(8):987–95. doi: 10.1007/s00284-017-1272-4 (PMC5486783; doi:10.1007/s00284-017-1272-4)
Supplement: Supplementary file 2 — Supplementary material 2 (DOC 31 kb) [file 284_2017_1272_MOESM2_ESM.doc]

Table S1. The occurrence of *B. longum* group in each gender.

Table S2. The occurrence of *B. breve* in each gender.
